# TRAJECTORIES OF CHRONIC PAIN AMONG OLDER VETERANS: IDENTIFYING PAIN-WORSENING PREDICTORS VIA MACHINE LEARNING

**DOI:** 10.1093/geroni/igae098.3909

**Published:** 2024-12-31

**Authors:** Chiyoung Lee, Yeri Kim, Seoyoung Kim, Beth Cohen, kent Kwoh, Hyochol Ahn, Juyoung Park, Heewon Kim

**Affiliations:** University of Arizona, Tucson, Arizona, United States; Soongsil University, Seoul, Republic of Korea; Soongsil University, Seoul, Republic of Korea; University of California, San Francisco, San Francisco, California, United States; University of Arizona, Tucson, Arizona, United States; University of Arizona, Tucson, Arizona, United States; University of Arizona, Tucson, Arizona, United States; Soongsil University, Seoul, Republic of Korea

## Abstract

Chronic pain is a major public health problem affecting approximately 100 million Americans and United States military Veterans, who constitute a particularly vulnerable group. While pain research in Veterans is actively underway, information on the longitudinal course of pain in this population is limited. This study aimed to 1) identify the various longitudinal pain status trajectories among older Veterans over a 10-year period and 2) detect factors predicting membership in the worsening trajectory of chronic pain. We analyzed data from 619 Veterans (mean age: 58.5 years) participating in the Mind Your Heart Study, an ongoing prospective cohort study examining diverse health outcomes among Veterans. Initially, we employed a generalized mixture model to identify pain trajectory classes using Brief Pain Inventory (BPI) pain intensity subscale score collected at 2-, 5-, and 10-year intervals. Two distinct trajectories were identified—low and high—both of which remained relatively stable. Subsequently, several feature selection methods extracted the predominant features from participants’ baseline characteristics that predicted membership in the high vs. low pain trajectory. These included: prior arthritis diagnosis; prior post-traumatic stress disorder (PTSD) diagnosis; depression symptoms; PTSD symptoms of avoidance, hyperarousal, and negative mood alterations; physical functioning; sleep quality; and overall health. The scikit-learn RandomForestClassifier, utilizing the refined feature set, achieved a classification accuracy of 0.79, yielding results nearly identical to those obtained using all 261 features. These findings are clinically informative and pertinent, highlighting potential intervention targets warranting intensive pain care plans based on probable long-term prognosis and discussing early treatment strategies among older Veterans.

